# Transgender and gender nonconforming people’s vulnerability to food insecurity: systematic review and meta-synthesis

**DOI:** 10.1590/0102-311XEN058825

**Published:** 2026-01-09

**Authors:** Igor Myron Ribeiro Nascimento, Ana Paula dos Reis, Renata Tannous Sobral de Andrade, Marcos Pereira

**Affiliations:** 1 Universidade Federal da Bahia, Salvador, Brasil.

**Keywords:** Food Security, Transgender Persons, Social Vulnerability, Social Stigma, Segurança Alimentar, Pessoas Transgênero, Vulnerabilidade Social, Estigma Social, Seguridad Alimentaria, Personas Transgénero, Vulnerabilidad Social, Estigma Social

## Abstract

Transgender, *travesti*, and gender nonconforming (TGNC) people are more vulnerable to food insecurity due to stigma and discrimination related to their gender identity. However, there is a lack of qualitative research on this topic. Therefore, this study examines the vulnerability and experiences of trans people with respect to food insecurity. A qualitative systematic review was conducted by searching seven electronic databases for studies in March 2024. Qualitative studies on the experience of food insecurity among trans people were included. The quality of studies was assessed using CASP, while the GRADE-CERQual approach evaluated the reliability of findings, which were then analyzed using meta-synthesis with thematic analysis. A total of 673 records were identified, of which five were selected. The most applied technique was semi-structured interviews. In total, four categories emerged from the meta-synthesis: vulnerability to food insecurity, experience of hunger, repercussions, and coping strategies. The results showed that TGNC people face high levels of stigma, discrimination, and victimization, which impact their food insecurity across different areas of life. Structural barriers, such as limited access to food and stable employment, exacerbate this insecurity, with negative consequences for physical and mental health. Factors such as skin color/ethnicity, gender, gender-based violence and lack of safe housing also contribute to this situation, including forced prostitution as a result of social barriers. Support networks such as the “chosen family” play an important role in reducing these effects. For trans people, the complexity of experiencing food insecurity is linked to transphobia, which creates barriers and violates the right to adequate food. They cope with hunger by activating support networks and seeking food assistance from institutions.

## Abstract (pt)

Pessoas transgênero, travestis e não conformes são mais vulnerabilizados ​​à insegurança alimentar devido ao estigma e à discriminação relacionados à sua identidade de gênero. No entanto, há uma escassez de pesquisas qualitativas sobre esse tema. Este estudo examina a vulnerabilidade e as experiências de pessoas trans com relação à insegurança alimentar. Uma revisão sistemática qualitativa foi conduzida por meio da busca em sete bases de dados eletrônicas em março de 2024. Estudos qualitativos sobre a experiência de insegurança alimentar entre pessoas trans foram incluídos. A qualidade dos estudos foi avaliada usando CASP, enquanto a abordagem GRADE-CERQual foi empregada para avaliar a confiabilidade dos resultados. Conduziu-se metassíntese com análise temática. Um total de 673 estudos foram identificados, dos quais cinco foram selecionados. A técnica mais aplicada foi a entrevista semiestruturada. Quatro categorias emergiram da metassíntese: vulnerabilidade à insegurança alimentar, experiência de fome, repercussões e estratégias de enfrentamento. Os resultados mostraram que as pessoas transgênero e não conformes com o gênero enfrentam altos níveis de estigma, discriminação e vitimização, que afetam sua insegurança alimentar em diferentes áreas da vida. Barreiras estruturais, como acesso limitado a alimentos e ao emprego estável, exacerbam essa insegurança, com consequências negativas para a saúde física e mental. Fatores como raça/etnia, gênero, violência de gênero e falta de moradia segura também contribuem para essa situação, incluindo prostituição forçada devido a barreiras sociais. Redes de apoio, como a “família escolhida”, desempenham um papel importante na redução desses efeitos. Para pessoas trans, a complexidade de vivenciar a insegurança alimentar está ligada à transfobia, que cria barreiras e viola o direito à alimentação adequada. Elas lidam com a fome ativando redes de apoio e buscando assistência alimentar através de instituições.

## Abstract (es)

Personas transgénero, travestis y no conformes con su género son más vulnerables a la inseguridad alimentaria debido al estigma y la discriminación relacionados con su identidad de género. Sin embargo, hay una falta de investigaciones cualitativas sobre este tema. Por lo tanto, este estudio examina la vulnerabilidad y las experiencias de las personas transgénero con respecto a la inseguridad alimentaria. Se llevó a cabo una revisión sistemática cualitativa mediante la búsqueda de estudios en siete bases de datos electrónicas en marzo de 2024. Se incluyeron estudios cualitativos sobre la experiencia de la inseguridad alimentaria entre las personas trans. La calidad de los estudios se evaluó utilizando el CASP, mientras que el método GRADE-CERQual evaluó la fiabilidad de los resultados y se analizó mediante una meta-síntesis con análisis temático. Se identificaron un total de 673 registros, de los cuales se seleccionaron cinco. La técnica más utilizada fue la entrevista semiestructurada. De la meta-síntesis surgieron cuatro categorías: vulnerabilidad a la inseguridad alimentaria, experiencia del hambre, repercusiones y estrategias de afrontamiento. Los resultados mostraron que las personas transgénero, travestis y no conformes con su género se enfrentan a altos niveles de estigmatización, discriminación y victimización, lo que afecta a su inseguridad alimentaria en diferentes ámbitos de la vida. Las barreras estructurales, como el acceso limitado a los alimentos y a un empleo estable, agravan esta inseguridad, con consecuencias negativas para la salud física y mental. Factores como la raza/etnia, el género, la violencia de género y la falta de una vivienda segura también contribuyen a esta situación, incluida la prostitución forzada debido a las barreras sociales. Las redes de apoyo, como la “familia elegida”, desempeñan un papel importante en la reducción de estos efectos. Para las personas trans, la complejidad de la inseguridad alimentaria está relacionada con la transfobia, que crea barreras y viola el derecho a una alimentación adecuada. Hacen frente al hambre activando redes de apoyo y solicitando ayuda alimentaria a las instituciones.

## Introduction

Globally, transgender, *travesti*, and gender nonconforming (TGNC) people endure complex and intersecting health inequities rooted in systemic stigmatization, discrimination, and the denial of basic rights. These structural barriers frequently result in broken family ties, housing insecurity, and exposure to street environments ^1^ ^,^ ^2^ ^,^ ^3^ ^,^ ^4^. These environments present ambiguous realities, ranging from offering welcome and survival strategies to exacerbating vulnerability ^5^ ^,^ ^6^ ^,^ ^7^ ^,^ ^8^. This reality raises questions about the effectiveness of government programs and policies to ensure social welfare. In this context, food insecurity emerges as a significant reality for TGNC and other vulnerable groups, particularly with regard to race and socioeconomic status.

Studies show that people who identify as TGNC have higher rates of food insecurity ^9^ ^,^ ^10^ ^,^ ^11^. In addition, people who belong to marginalized groups, such as African Americans, Latinos, and low income people, may be even more affected by food insecurity, requiring prioritized support and additional safety nets to meet their basic needs ^11^ ^,^ ^12^ ^,^ ^13^.

The overlap of different forms of stigma, rooted in broader systems of inequality and power, has led researchers to consider different approaches to its study and analysis. This is crucial as TGNC people living in regions with high rates of food insecurity, combined with sociopolitical contexts that perpetuate discrimination and stigma, face particularly high risks of food insecurity ^14^. Given stigma’s connection to historical and contemporary manifestations of inequality, power, and systems of domination, intersectionality emerges as a promising theoretical approach to studying stigma ^15^.

Food security means that “*all people, at all times, have physical, social and economic access to sufficient, safe and nutritious food that meets their dietary needs and food preferences for an active and healthy life*” ^16^ (p. 3). This definition recognizes a degree of subjectivity in food security, such that individual perceptions of adequate food quantity and quality may vary by race/skin color, gender, and class.

The 2023 global assessment of the Food and Agriculture Organization of the United Nations (FAO) ^17^ highlights persistent challenges, particularly regarding hunger. From 691 to 783 million people were affected by hunger in 2022, representing about 29.6% of the world’s population, with the African continent facing the highest proportion of food insecurity. Adult women in rural areas are at even greater risk of food insecurity due to loss of employment, income, and unpaid responsibilities. Although other gender and race/skin color identities are not specifically mentioned, it is important to recognize that they may also face significant food insecurity challenges. This persistence underscores the continued need for global efforts to address the issue in a more inclusive and comprehensive manner.

This study is motivated by the lack of systematic reviews that address food insecurity among TGNC people. Most studies on gender relations and food insecurity focus on cisgender perspectives ^18^ ^,^ ^19^ ^,^ ^20^ and/or are epidemiological studies ^11^ ^,^ ^12^ ^,^ ^21^ ^,^ ^22^. This meta-synthesis interprets and synthesizes data across studies to extract meaningful insights, generate new knowledge on how gender identity shapes vulnerability to food insecurity, and explore lived experiences and coping strategies related to food insecurity. Thus, the aim of this study is to understand the vulnerability and experiences of TGNC people with respect to food insecurity.

## Methods

A qualitative systematic review with meta-synthesis ^23^ was conducted according to the Cochrane Qualitative and Implementation Methods Group ^24^. The review question was “What are TGNC people’s experiences of food insecurity?”.

### Eligibility criteria

Qualitative or mixed studies that analyzed vulnerability to and experiences of food insecurity among trans people were eligible. Thus, experience is understood as an interpretive process that connects the individual with their culture, social relations, and historical context ^25^. No publication date or language restriction was set. Studies that focused on biomedical aspects related to hormonalization, sexual reassignment, and industrial silicone use were excluded, as were those resulting from quantitative or mixed research without an in-depth epistemological examination of the issue.

### Search strategy

The search strategy was applied in the following electronic databases: Web of Science, MEDLINE via PubMed, Embase, LILACS, APA PsycInfo, CINAHL via EBSCO, and Google Scholar for the grey literature. In addition, the bibliographic reference lists of relevant studies were examined to identify potentially eligible ones. The searches were conducted in November 2023 and updated in March 2024.

The searches used *Medical Subject Headings,* Embase keywords, *Health Sciences Descriptors*, and Thesaurus terms to identify references in these databases (Supplementary Material - Table S1; https://cadernos.ensp.fiocruz.br/static//arquivo/suppl-e00058825_9777.pdf). These keywords were combined with the Boolean operators “OR” and “AND”, and their entry terms in all databases.

### Screening process and data extraction

The screening process was conducted using Rayyan (https://www.rayyan.ai/). The initial screening included identification and exclusion of duplicates, reading of titles and abstracts, and application of eligibility criteria by the reviewers I.M.R.N. and M.P. For those studies in which there was disagreement, a third reviewer, R.T.S.A., was consulted.

The following information was extracted from the studies: identification, authors, country, year of publication, place of study, interlocutors, ethnic identity, epistemology, analyses, methods, and food insecurity measurement.

### Quality appraisal

The quality of the studies was assessed using the *Critical Appraisal Skills Programme* (CASP), with 10 domains. Qualitative studies were classified into two categories: “A” for high methodological rigor, fulfilling at least nine of the 10 items; and “B” for moderate methodological rigor, fulfilling at least five of the 10 items ^26^ ^,^ ^27^. The maximum score was 10 ^26^.

The GRADE-CERQual (https://www.cerqual.org/) was used to assess the reliability of the results, considering four main components: the methodological limitations of the studies; the coherence of the review’s conclusions, verifying the alignment between primary study data and the overall conclusion; the adequacy of available data to support the conclusion; and finally, the relevance of the studies to the review question. Based on this analysis, a level of confidence to the evidence was assigned, classifying it as high, moderate, low, or very low, which enabled the assessment of the soundness of the review’s conclusions ^28^ ^,^ ^29^.

### Strategy for data synthesis

The studies were analyzed in depth using thematic analysis to identify recurring patterns. Key categories were systematically extracted and linked to participants’ narratives, with particular attention to the intersections between food insecurity and TGNC people identity. This approach illuminated how stigma and structural vulnerability create barriers to accessing basic rights, including adequate nutrition ^30^. Additionally, the analysis explored potential intersectional relationships among stigma, vulnerability, and hunger.

### Stigma and hunger vulnerability from an intersectional perspective

Stigma is a crucial concept for discussing social organization and its relationship with subjects. It is a social process that is produced and contextualized, reflecting a multifaceted and historical nature. It plays a fundamental role in the production and reproduction of relations of power and control in all social systems ^31^ ^,^ ^32^. Thus, it is a broad system that produces and reproduces inequalities, both for groups and subjects who benefit from it and for those marginalized within this organization. As such, these “discursive technologies” ^1^ ^,^ ^33^ ^,^ ^34^, articulate a web guided by “necrobiopower” ^35^, through an interconnected network that directs how subjects and groups navigate the system and its agents of action within the social structure.

As a result, challenging the naturalness of cisnormativity - a normative model of “being” that functions as a regulatory fiction, institutionalizing socially legible identities and associating them with essentialist notions of “normality”, “sanity”, and “biological naturalness” ^7^ - within society results in the emergence of stigma as a mechanism of social control. In this way, the process of stigmatization deepens situations of food scarcity and contributes to a greater prevalence of food insecurity, diets based on ultra-processed foods and difficulties in accessing adequate nutritional support networks ^21^.

Vulnerability, in turn, is “*the set of individual and collective aspects related to the degree and form of exposure to a given situation and, inseparably, to greater or lesser access to adequate resources to protect oneself from the undesirable consequences of that situation*” ^36^ (p. 7; our translation). Understanding it involves an intersection of contextual factors such as race, gender, social class, nationality, generation, and sexuality ^37^. Therefore, in order to analyze it, it is essential to consider three components: individual (quality of information and its use), social (interaction between information and available resources), and programmatic (policy integration of government action with the specific needs of vulnerable groups) ^36^ ^,^ ^37^ ^,^ ^38^ ^,^ ^39^.

In this context, stigma and vulnerability are clearly influenced by structural elements that organize society, such as racism, patriarchy, and class relations. These intersections are key to understanding how stigma contributes to the vulnerability and inequality of specific groups, such as TGNC people ^31^ ^,^ ^40^. Understanding these interconnections is crucial to interpreting the causes of hunger and recognizing how public policies often fail to address the needs of bodies beyond cisnormativity.

## Results

### Search results and study selection

**Figure 1:**
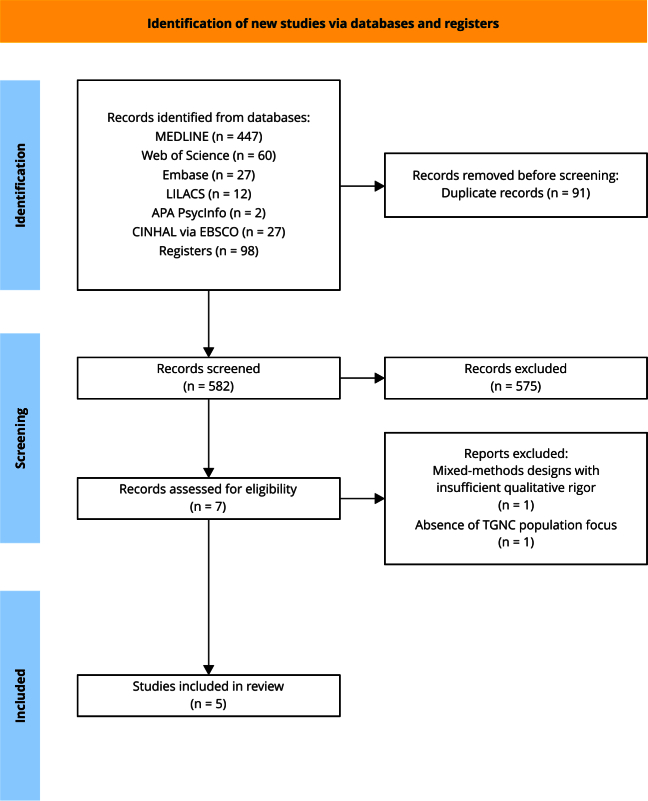
Study selection flowchart.

A total of 673 records were identified, of which seven were selected to assess eligibility, and five were included in the meta-synthesis. The exclusion criteria included a mixed-methods design with insufficient qualitative rigor (n = 1) and the absence of a TGNC population focus (n = 1) (Figure 1).

TGNC: transgender, *travesti*, and gender nonconforming. Source: prepared by the authors.

### Characterization and qualitative synthesis

The studies were published from 2019 to 2024 (Box 1; Supplementary Material - Table S2; https://cadernos.ensp.fiocruz.br/static//arquivo/suppl-e00058825_9777.pdf), four in the United States and one in Canada. A total of four studies examined the relationship between hunger or food insecurity and the TGNC population, most of which were conducted in university settings. A fifth study examined this relationship in the context of HIV among cisgender and transgender women. Semistructured interviews were widely used, with only one study employing the photovoice technique. Research methods varied, including thematic analysis, constructivism, a socioecological perspective, and ethnographic and community-based qualitative approaches.

**Box 1:** Characteristics of included studies.

ID	Study (Year)	CASP	Country	Interlocutors	Ethnic identity	Epistemology	Analysis	Methods	Food insecurity measurement
E1	Kirby & Linde ^42^ (2020)	9-A	United States	26 students who identified as TGNC: Transfemale/transwoman 1 Male 2 Gender queer/gender nonconforming 6 Transmale/transman 7 Different identity 10	American Indian/ Native Alaskan 1 Black/African American 1 Asian or Pacific Islander 2 Hispanic or Latino 4 White/Caucasian 22	NA	Systematic thematic analysis	Interviews and surveys	*Rainbow Health Initiative’s Voices of Health Survey* (VHS)
E2	Russomanno et al. ^14^ (2019)	10-A	United States	20 TGNC people: Gender nonconforming 1 Gender fluid 2 Gender queer 2 Transgender female 3 Nonbinary 4 Transgender male 8	NA	Minority stress theory	Paradigmatic framework of constructivism	Semistructured telephone interviews	*USDA Food Security Survey Module*
E3	Sernick et al. ^46^ (2022)	10-A	Canada	64 WLWH: Heterosexual female 1 Gender queer 1 Women 2 Transgender 3 Two-spirit 3 Cisgender women 54	Mixed ethnicity or other visible minorities 5 Black/African 9 White 19 Indigenous 31	Socioecological framework	Iterative thematic analysis	Semistructured interviews	Interview questions
E4	Henry et al. ^45^ (2023)	8-A	United States	22 LGBTQIA+ students: Bigender 1 Gender fluid 1 Transgender 2 Male 3 Nonbinary 8 Female 9	Black 1 Latinx 4 Asian and Pacific Islander 4 White 13	NA	Ethnographic approach	Semistructured interviews	Self-reported
E5	Lumens et al. ^41^ (2024)	8,5-A	United States	8 self-identified LGBTQIA2S+ university students: Cisgender and/or female 5 Other 3	White 5 Non-white 3	Intersectionality and queer theories	Stenner’s thematic decomposition analysis	Photovoice and semistructured interviews	Self-reported

CASP: *Critical Appraisal Skills Programme*; NA: not available; TGNC: transgender, *travesti*, and gender nonconforming; USDA: U.S. Department of Agriculture; WLWH: women living with HIV.

### Methodological quality

The assessment of methodological rigor according to the CASP criteria showed that three of the articles were classified as A (high methodological rigor). Ethical research procedures that were not explained in the study methodology and a lack of explicit interaction between the researcher and participants in the field were the aspects that scored negatively and contributed to moderate methodological rigor (Box 1; Supplementary Material - Table S3; https://cadernos.ensp.fiocruz.br/static//arquivo/suppl-e00058825_9777.pdf).

The GRADE-CERQual assessment revealed a moderate level of confidence in the reliability of the findings, primarily due to the methodological soundness and coherence of the results. However, limitations were identified regarding adequacy and relevance, stemming from the scarcity of data available for a deeper understanding of the studied realities and their socio-historical context, particularly in countries from the Global North (Box 2).

**Box 2:** Reliability of the included studies.

FINDINGS	STUDIES	METHODOLOGICAL LIMITATIONS	COHERENCE	ADEQUACY	RELEVANCE	CERQual ASSESSMENT OF CONFIDENCE IN THE EVIDENCE
Vulnerability to food insecurity						
Vulnerability to food insecurity was identified through three subthemes: the process of gender reassignment; transphobia and its impact on employability, family exclusion and access to social assistance; and finally, gender risks and violence in social spaces	E1, E2 & E5	There were no concerns about the methodological limitations of the studies, as they were all adequately described according to CASP and COREQ	No or very little concern about coherence	No or very minor concerns about adequacy that are unlikely to reduce confidence in the review finding	Moderate concerns about the relevance of the studies being conducted in only two high-income countries with similar development contexts	High confidence
Experiences						
Trans people reported financial challenges accessing quality food in sufficient quantities, mainly due to complex employment situations. Many of them mentioned that it is easier to buy ultra-processed food due to budget constraints, and some even resort to leftovers and scraps. These financial difficulties suggest that the food insecurity faced by trans people is more related to economics than to the lack of food availability	E1, E2, E3 & E4	There were no concerns about the methodological limitations of the studies, as they were all adequately described according to CASP and COREQ Minor or moderate concerns	No or very little concern about coherence	No or very minor concerns about adequacy that are unlikely to reduce confidence in the review finding	Moderate concerns about the relevance of the studies being conducted in only two high-income countries with similar development contexts	High confidence
There is often a competition between monthly financial obligations, which include expenses such as rent, electricity, basic sanitation, and the costs associated with the process of body feminization/masculinization. As a result, food is often not given the priority it deserves	E2 & E4	There were no concerns about the methodological limitations of the studies, as they were all adequately described according to CASP and COREQ	No or very little concern about coherence	Minor or moderate concerns about the reliability of the review finding because no other studies confirm the finding	Moderate concerns about the relevance of the studies being conducted in only two high-income countries with similar development contexts.	Moderate confidence
In the context of accessing food assistance, shelter, and other resources, trans people highlighted physical challenges related to food preparation, such as lack of adequate space and essential equipment	E3, E4 & E5	There were no concerns about the methodological limitations of the studies, as they were all adequately described according to CASP and COREQ	No or very little concern about coherence	No or very minor concerns about adequacy that are unlikely to reduce confidence in the review finding	Moderate concerns about the relevance of the studies being conducted in only two high-income countries with similar development contexts	High confidence
Trans people share their experiences of the challenges of risk and insecurity when accessing food assistance services. They express the need for more exclusive welcoming spaces for this population to reduce feelings of insecurity when sharing these spaces with, for example, cisgender men	E2, E3, E4 & E5	There were no concerns about the methodological limitations of the studies, as they were all adequately described according to CASP and COREQ	No or very little concern about coherence	No or very minor concerns about adequacy that are unlikely to reduce confidence in the review finding	Moderate concerns about the relevance of the studies being conducted in only two high-income countries with similar development contexts	High confidence
Repercussions of food insecurity						
Food insecurity among trans people can have a significant impact on health, both physical and mental. In addition, food insecurity can contribute to the development of new health problems and aggravate existing ones. Physically, it can manifest itself in symptoms such as nausea, loss of appetite, weight gain, hypercholesterolemia, nutritional deficiencies and hypertension. In terms of mental health, it can trigger depression, anxiety, stress and eating disorders	E1, E2 & E4	There were no concerns about the methodological limitations of the studies, as they were all adequately described according to CASP and COREQ	No or very little concern about coherence	No or very minor concerns about adequacy that are unlikely to reduce confidence in the review finding	Moderate concerns about the relevance of the studies being conducted in only two high-income countries with similar development contexts	High confidence
Coping strategies						
Resilience was identified as a strategy to mitigate the impact of food insecurity on trans people. This includes the use of techniques such as meditation and breath control, as well as maintaining a positive outlook in the face of adversity, aiming to strengthen the ability to face and overcome these challenges	E2	There were no concerns about the methodological limitations of the studies, as they were all adequately described according to CASP and COREQ	No or very little concern about coherence	Minor or moderate concerns about the reliability of the review finding because no other studies confirm the finding	Moderate concerns about the relevance of the studies being conducted in only two high-income countries with similar development contexts	Moderate confidence
Community resilience through support networks manifests itself in mutual collaboration to develop strategies to address hunger. This may include direct financial support, provision of resources, or participation in social and food assistance programs	E2 & E4	There were no concerns about the methodological limitations of the studies, as they were all adequately described according to CASP and COREQ Minor or moderate concerns	No or very little concern about coherence	Minor or moderate concerns about the reliability of the review finding because no other studies confirm the finding	Moderate concerns about the relevance of the studies being conducted in only two high-income countries with similar development contexts	Moderate confidence

CASP: *Critical Appraisal Skills Programme*; COREQ: *Criteria for Reporting Qualitative Research*.

### Meta-synthesis

**Figure 2:**
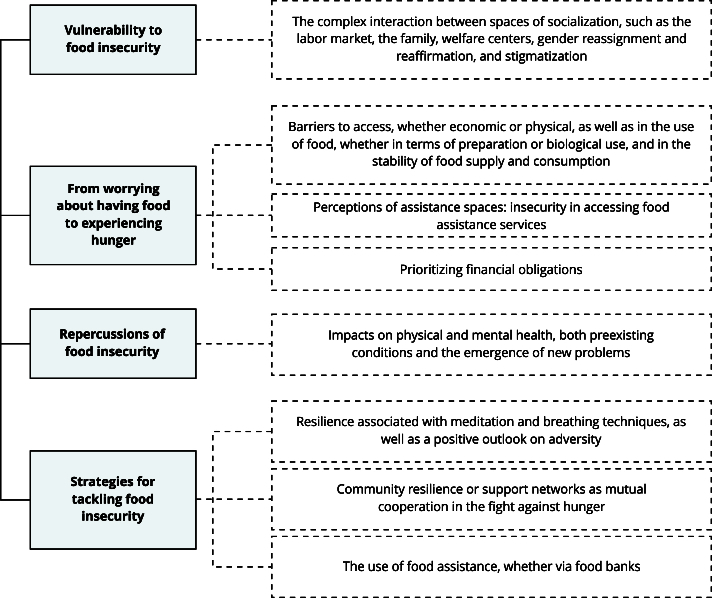
Thematic map of seq-derived findings in analytical categories.

Source: prepared by the authors.

The thematic analysis identified four categories: vulnerability to food insecurity, the experience of hunger, the repercussions of food insecurity, and coping strategies, which are detailed as follows. At the end of the analysis, eight main findings were identified (Figure 2).

#### Issue 1: vulnerability to food insecurity

Vulnerability to food insecurity was identified through three subthemes: the process of gender reassignment; transphobia and its implications for employability, family exclusion and access to social assistance; and finally, gender risk and violence in spaces of socialization.

(1.1) Gender reassignment emerges as a process of constructing an understandable identity, both in terms of self-perception and the perception of others. Through this process, mind and body work together to reduce the likelihood of violence, whether physical or symbolic:

“(...) *I think for many people it absolutely could be harmful. Being a straight-passing man in a straight relationship, I do have a lot of the privilege of not experiencing a lot of the exploitation that other queer folks go through... I’m very lucky to say and privileged to say that my food insecurity is not impacted by my queerness*” (queer people) ^41^ (p. 118).

“*I try to be positive and see a body that I love and value... On bad days I see a body that’s sluggish and grotesque. One that can never pass for a woman*” (transgender female) ^42^ (p. 36).

In this process of adapting the body, there is a particular trajectory among black TGNC people. Although adaptation can be seen as a protective mechanism or as a defining component of what is conceived as a “*true transsexual*” ^43^, from a biomedical perspective it is shaped by racialization, which manifests racism and results in specific violence, with severe consequences for the self-perception and body image of these people:

“*The world tells me that I’m not a real trans person unless I take hormones and get surgery. And then even if I do these things, being trans isn’t exactly what the world extolls as beautiful. Also, as a POC* ENT#091;person of colorENT#093;*, I don’t often see people who look like me in the media. I have little positive representation or role models to look up to, so I don’t know what good body image for someone like me looks like*” (transgender female) ^42^ (p. 36).

(1.2) Gender discrimination or transphobia, as forms of stigmatization, vulnerability, and generators of inequalities, have a significant impact on various social spheres and institutions. This results in limited access to food services and employment opportunities, as well as the breakdown of kinship relationships:

“*We had a couple food banks near us, but we were iffy about going to them. Pretty much all of the things in our area are run out of conservative churches, so my wife would have to wear a binder and put her hair up. I’d also have to dress more feminine than I was comfortable with at that time and just keep our heads down, hope nobody noticed anything off*” (nonbinary) ^14^ (p. 94).

“*And considering that the only thing that inherently changed is like ‘hi, I’m trans now,’ you kind of have to figure... With the lost economic opportunity of being unemployed for an extended period of time, obviously, that has an impact on my ability to access food*” (transgender female) ^41^ (p. 119).

#### Issue 2: from worrying about having food to experiencing hunger

For TGNC individuals, food insecurity can manifest as a complex intersection of barriers, where factors such as social stigma, lack of inclusion and discrimination in work environments contribute to food vulnerability. In this sense, the analysis of subthemes considered the dimensions of food security. Economic and physical access to food stands out, encompassing not only the ability to acquire food but also to do so in a socially acceptable manner, considering factors such as price and “competition” with other basic needs. Reflecting on the use of food in relation to health and cultural issues accentuates the importance of understanding not only nutrition but also the social and cultural role of food ^44^. Another subtheme involves the experience of socialization and support spaces, emphasizing how TGNC individuals’ pursuit of social support and assistance directly impacts their food security access.

(2.1) It is clear from their statements that TGNC people face financial challenges in accessing adequate amounts of quality food. They mentioned that due to budget constraints, they often have to resort to buying ultra-processed and/or processed foods that are considered inexpensive. They also report seeking alternatives, such as using leftovers and scraps from meals or selling personal items to raise money. This reality underscores the need to address the economic barriers that directly impact their diets:

“*I can get a can of ravioli for 85 cents, and that’s a full meal. A head of lettuce or broccoli costs $2+ and I need other ingredients to create a meal*” (not reported) ^42^ (p. 35-6).

“(...) *I think that is sort of included in the food insecurity is access to food that is good for you. One of my coping tactics is to eat food that’s cheap but bad for me*” (white nonbinary) ^45^ (p. 126).

“*We have dived dumpsters throughout the years many times, which usually produce something good, but most of the time, dumpsters are locked up*” (transgender male) ^14^ (p. 94).

(2.2) There is often a “competition” between monthly financial obligations, which include expenses such as rent, electricity, and car maintenance, and the costs associated with the process of body feminization or masculinization. As a result, taking care of food often seems to take a back seat:

“*I’m gonna have to have another surgery. I was hoping that maybe I wouldn’t, but it’s becoming apparent to me that I need to. I hate buying food because every time I buy food I think about how it’s getting - it’s something I need, but it’s getting in the way of something else that I need*” (transgender male) ^14^ (p. 94).

“*I’m on hormone replacement therapy. So sometimes I feel like when I ask someone, like my mom, or when I’m just running low on money, I feel like I’m expensive because I’m paying for hormones. So, whenever I run out, that’s another $30 that I have to spend, and I feel like if I weren’t the way I was, then I would have that $30 for food, rent, or whatever*” (Latinx transgender male) ^46^ (p. 6).

(2.3) In this scenario, Sernick et al. ^46^ introduces the discussion of the cultural dimension of food security by pointing out that interlocutors reported lack of variety in foods, especially those traditionally associated with a particular culture, such as Indigenous peoples.

(2.4) Challenges were reported regarding the physical spaces where participants store, prepare, and consume food. They mentioned a lack of appropriate spaces and essential equipment, such as functional kitchens and adequate storage areas:

“*I do wish that I could get into better housing, right. Something with, um, a kitchenette or something with a kitchen*” (transgender Indigenous woman) ^46^ (p. 6).

“*What* ENT#091;my momENT#093; *brings is limited by the amount that I can store in my refrigerator and freezer. I can’t just simply buy a bigger appliance because each one is at the size limit for what is allowed on campus. I had to obtain accommodations and doctor’s notes just to have a separate freezer*” (not reported) ^41^ (p. 123).

(2.5) The search for food assistance for TGNC people presents complex challenges. Although the need for food is universal, barriers such as inadequate services and stigmatizing environments keep many people away from these spaces. One participant expressed fear of receiving pitying looks or invasive questions about their identity: “*There are times when I don’t want to seek help because I’m worried* (...) *they might ask me, you know, what do I identify as...*” (white bisexual transgender female) ^45^ (p. 129). Another participant pointed out how the strong presence of religious symbols in places of support can create discomfort and increase distance from the LGBTQ+ community: “*it is still nearly impossible to avoid Christian-affiliated symbols.* (...) *the close proximity of policy to Baptist influence... can further distance LGBTQ+ people*” (transgender female) ^45^ (p. 122). These experiences reveal the complexities and barriers that TGNC people face in accessing essential resources, and highlight the need for support spaces that are more inclusive and sensitive to their realities.

#### Issue 3: repercussions of food insecurity

Regarding the repercussions of food insecurity, subthemes related to its impact on the physical and mental health of TGNC people were identified.

(3.1) Physically, this situation can manifest as weight gain: “ENT#091;I wasENT#093; *eating really unhealthy food, because it was cheaper. Gained weight, and felt sick and tired all the time*” (transgender male) ^14^ (p. 95). It can also manifest as nutritional deficiencies due to inadequate consumption of ultra-processed foods: “*I feel like a lot of times I was eating things that weren’t very nutritious, because I could get ‘em for $0.99.* (...) *They’re not giving me the right protein and vitamins.* (...) *I was sick all the time*” (gender fluid) ^14^ (p. 94).

(3.2) In terms of mental health, experiencing food insecurity can trigger depression, anxiety, stress, and eating disorders. One participant reported that constant hunger made him depressed and stressed, negatively affecting his body and well-being: “*I was hungry 24/7. No one feels good when they’re hungry* (...) *and your body isn’t being given the right nutrition*” (gender fluid) ^14^ (p. 95). Another participant highlighted the importance of nutrition services sensitive to gender dysphoria and the community’s history of eating disorders: “*I’m very worried that they wouldn’t be sensitive to my eating disorder and also not understand that my eating disorder stems from gender dysphoria and that comes with its own unique set of challenges that I need to recover from*” (not reported) ^42^ (p. 37).

This lack of psychological support exacerbates the cycle, leading to the use of energy to cope with emotional demands: “*I feel like a lot of it is my mental health putting me here. I think sometimes that, along with food insecurity, can be so intertwined because it’s such a cycle*” (white nonbinary) ^45^ (p. 126).

#### Issue 4: strategies to address food insecurity

In analyzing strategies to address food insecurity, three subthemes were identified: individual resilience to mitigate hunger; community resilience through the creation of support networks; and food assistance services such as food banks, community restaurants, income transfers, and others.

(4.1) Individual resilience was identified as a strategy to mitigate the impact of food insecurity on trans people. This includes maintaining a positive outlook in the face of adversity. This approach aims to strengthen the ability to face and overcome the challenges of food insecurity: “*I guess we’re both pretty resilient.* (...) *We just try to continue to be positive and hope that she’ll get a job and now she has one luckily. We just try to remain positive and not let that affect her son and things like that, but we don’t always eat*” (genderqueer) ^14^ (p. 95).

(4.2) Community resilience, expressed through support networks, materializes in mutual collaboration to develop strategies to address hunger. This collaboration may include direct financial support, the provision of resources, or participation in social and food assistance programs. This solidarity-based interaction highlights the importance of the community as a vital resource in mitigating the effects of food insecurity:

“*I feel like kind of working through these issues together and being there for each other kind of immediately at low points in our life has created this bond and definitely kind of shown each other our true colors right from the get go. I think, in a way, it’s made our relationship really strong...*” (gender fluid) ^14^ (p. 96).

“*Giving us more resources to access mental health professionals could go a long way in helping with food insecurity. If we know how to deal with stress and anxiety, you know, if we have more coping mechanisms, I feel like this would go a long way to improving our overall health*” (white asexual nonbinary) ^45^ (p. 129).

(4.3) Food assistance emerges as a strategy that not only guarantees access to necessary food: “*The food pantry represents one of the most readily available sources of cheap, healthy food for me. It’s within walking distance, and I can carry what I get back*” (queer and two-spirit) ^41^ (p. 120), but also promotes social identification when implemented inclusively, as one participant noted: “*...they were a queer-affirming church, so that was the main reason I even felt comfortable going*” (white nonbinary) ^44^ (p. 129).

## Discussion

The articles analyzed in this review demonstrate the barriers that TGNC people face in adopting a healthy diet, which is inextricably linked to the principles of food safety ^14^ ^,^ ^41^ ^,^ ^42^ ^,^ ^45^ ^,^ ^46^. Understanding a healthy diet goes beyond the mere ingestion of nutrients and encompasses eating practices imbued with sociocultural meanings. Food is not just a source of nutrients; it carries cultural, behavioral, and affective meanings ^44^ ^,^ ^47^. Therefore, promoting health through food should consider food as a source of pleasure and cultural and familial identity ^48^.

In addition, dietary diversity is critical because no single food can provide all the nutrients needed for a complete diet and good health. This underscores the importance of considering not only nutritional aspects but also cultural and individual contexts when promoting healthy eating ^48^.

In light of these issues, the selected articles reveal issues related to food monotony and food deserts ^49^ ^,^ ^50^. They identify a dietary pattern related to the unequal distribution of food, resulting in increased consumption of ultra-processed foods to the detriment of fresh or minimally processed foods. They also point to a tendency to use refined products, canned goods, sausages and, in some cases, products of questionable hygienic and health quality.

A healthy diet is fundamental to maintaining good health ^17^ ^,^ ^48^. However, it has been recognized that there are structural and sociocultural factors that intertwine and compromise well-being. These factors include poverty, the effects of colonialism ^44^ ^,^ ^51^ and dislocation from traditional foods ^17^ ^,^ ^48^, the agri-food system ^17^ ^,^ ^48^, immigration experiences ^17^ ^,^ ^44^, histories of gender-based violence, and racialization ^44^ ^,^ ^51^ ^,^ ^52^.

A study that examined food insecurity among LGBTQ+ adults during the COVID-19 pandemic in the United States found that trans adults faced significant disadvantages in terms of socioeconomic status, employability, and social protection ^11^. It also found that being trans not only increased the likelihood of unemployment but also contributed to a higher likelihood of food insecurity. Moreover, African American ethnicity was a significant predictor of food insecurity among LGBTQ+ adults during the pandemic. These findings are consistent with other research conducted in the United States, which also found that non-white people in the LGBTQ+ community were more likely to experience food insecurity ^22^. For example, food insecurity was more prevalent among trans and African American people, highlighting the role of racism and gender discrimination in this phenomenon. These studies corroborate the findings of the reviewed studies by demonstrating how these factors affect TGNC citizenship and also address the importance of race/ethnicity in the reported experiences, illustrating the role of intersectional analysis of food insecurity among TGNC people.

Studies of TGNC university students have shown a greater presence of food insecurity ^9^ ^,^ ^10^, especially when compared to cisgender students ^13^. This complex relationship between transgender identity and food insecurity reveals the experience of hunger as a result of stigma, social vulnerability, and structural barriers, and underscores the critical importance of support networks to mitigate the effects of social stigma in this group.

A systematic review in Brazil ^53^ that addressed socio-racial inequalities as a significant factor in food insecurity highlighted the association of food insecurity with a history of being located in areas with many migrants and the descendants of black slaves, such as *quilombolas*. This corroborates the findings of this review, as most of the identified statements discussed the financial and housing instability experienced by TGNC people.

These results echo similar findings from studies conducted in the United States and Canada that linked food insecurity in the TGNC community to factors such as poverty, the historical impact of colonialism, racism, sexism and gender violence, including transphobia ^13^ ^,^ ^44^. Given that people with underrepresented identities in terms of class, ethnicity, or sexual orientation face a disproportionate burden of food insecurity ^52^, this scenario elucidates the importance of intersectional analysis in understanding and proposing effective interventions in the face of these social inequalities. This means recognizing the complex interplay between different forms of marginalization, enabling more comprehensive approaches that are sensitive to the specific needs of each affected group.

The body and gender performance emerge as essential discursive elements for understanding the challenges faced by TGNC people. (Pre)discursive formations function as apparatuses that manufacture social imaginaries, simultaneously constructing the ontological boundaries of “otherness” while prescribing the socio-spatial positions these othered subjects may occupy. This signifying practice constitutes a fundamentally political operation: through its meaning-making regimes about alterity, it reifies hierarchical relations and legitimates structural violence under the ideological guise of normative compliance. Thus, violence operates not as an aberrant phenomenon, but rather as a constitutive technology of political ontology, a disciplinary mechanism that materializes the biopolitical project of demarcating between grievable and ungrievable lives ^1^ ^,^ ^33^ ^,^ ^34^.

Thus, the analysis of “body image” in relation to eating disorders and food insecurity reveals the significant influence of the cis-heteronormativity on self-image, especially considering the processes of reassignment ^1^ ^,^ ^42^. In this sense, some trans people may resort to identity concealment strategies to avoid microaggressions and explicit acts of discrimination ^43^. This phenomenon exemplifies the materialization of (pre)discursive violence.

In addition, the forced prostitution experienced by TGNC people adds complexity to this scenario, highlighting the multiple barriers of transphobia that scar their bodies and even affect their priorities in monthly spending ^14^ ^,^ ^41^ ^,^ ^42^ ^,^ ^45^ ^,^ ^46^. Issues such as gender identity, class, ethnicity, among others, play a crucial role in shaping food insecurity, the emergence of eating disorders, and self-image issues within this population. This analysis reveals the modern-colonial gender system ^54^ as an apparatus of normative violence that operates simultaneously at macrostructural and biopolitical levels. Functioning through what is termed the “coloniality of gender”, this regime: disciplines bodies through cis-heteropatriarchal grammars of being; forecloses existential possibilities for gender-dissident subjects; and perpetuates what is identified as the “coloniality of power” ^55^ through gendered epistemologies of exclusion. The intersection of these aforementioned analytical vectors demands radical epistemic disobedience: only through decolonial intersectionality can we both comprehend the necropolitical realities facing trans communities and construct liberatory frameworks that dismantle these oppressive architectures ^33^ ^,^ ^54^ ^,^ ^55^.

In this way, gender stigma, especially in the context of trans people, becomes a significant barrier to access to stable employment opportunities, adequate health, and nutrition. If there is no formal, stable source of income, how can there be adequate access to food? The gender discrimination experienced by TGNC people therefore plays a crucial role in the development of food insecurity in this group.

In this context, stigma emerges as a central element in access barriers for TGNC people, particularly concerning income, which plays a critical role in the search for adequate food ^14^ ^,^ ^31^ ^,^ ^42^ ^,^ ^45^ ^,^ ^46^. People who are stigmatized may experience food insecurity and resort to strategies considered “socially unacceptable” to obtain food, such as seeking charitable assistance, exchanging sex for food, or engaging in illicit activities ^56^ ^,^ ^57^ ^,^ ^58^ ^,^ ^59^. In addition, a scoping review ^58^ demonstrated the role of forced prostitution as a response to the violation of personal dignity and autonomy, which poses significant risks. These survival strategies reflect the complexities and challenges faced by marginalized groups in meeting their basic needs, and underscore the need for sensitive approaches to addressing inequalities associated with food insecurity.

In this sense, it is possible to identify a veritable necropolitical apparatus that acts upon TGNC existences, operating via systematic techniques of promoting death, both on the symbolic level (denial of identities, pathologization, discursive erasure) and on the material level (physical, economic, and legal violence). This regime of necrobiopower ^35^ not only classifies which bodies are worthy of mourning and which can be discarded, but also actively structures the conditions that deny TGNC people even minimal recognition of their humanity. Thus, a hierarchy of lives is consolidated in which some are naturalized as deserving of protection and rights, while others are destined for social elimination.

Studies show that forced prostitution is a reality in the lives of trans people, whether to provide food or basic necessities, or for gender reassignment processes ^14^ ^,^ ^45^ ^,^ ^46^. For example, during the pandemic in Brazil, a study found that 20.2% of transgender women and travestis faced some degree of food insecurity, which affected purchasing power, dietary diversity, and access to healthy food. The study also found gender prejudice, rejection at work, and frequent physical violence ^21^. This highlights the pernicious role of the lack of opportunities for trans people, which often forces them into sex work.

Gender-based violence, as a sociocultural and psychological process, also emerges as an important barrier to food security. The results of this review confirm the observation that many TGNC people avoid going to care settings due to fear of violence, both from other users and from professionals ^14^ ^,^ ^42^; and corroborate similar findings among sex workers ^60^. Daily experiences of violence, with partners, family members, and in care settings, reveal gaps in care perspectives that need to address the structural issues that affect the care process. The impact of social and psychological factors on the dynamics of food insecurity is therefore evident.

In addition, food insecurity can be associated with sexual health risk situations, including HIV infection and other sexually transmitted infections, revealing its multifaceted nature as a cause, consequence, and aggravating element of a given situation ^56^ ^,^ ^59^ ^,^ ^60^. Thus, it becomes clear that food insecurity is part of a complex intersection of social, cultural, physiological, and psychological factors, with impacts that both contribute to and are caused by this condition.

In line with this perspective, a conceptual model was developed in an attempt to understand the relationship between food insecurity and health through the lived experience of African American women in the United States. This model distinguishes between two types of hunger experience: the physical experience (hunger of the body) and the emotional experience (hunger of the mind) ^61^. The former is related to the lack of economic and physical resources to access food, while the latter refers to food deprivation resulting from anxiety caused by social problems, such as poverty and violence, and health problems. Although these experiences are interrelated, “hunger of the mind” was more prominent, as past violence, depression, and physical and mental illness were more prominent in the narratives of the participants approached by the authors. This discussion contributes to the understanding of issues related to the emergence of eating disorders among TGNC people, as well as other physical and mental effects, as found in the studies included in this review.

Housing insecurity, which is mainly the result of financial insecurity and interpersonal rejection, coupled with stigma, discrimination, and changes in the housing landscape, leads to psychological tension and risky behaviors ^4^ ^,^ ^13^. The lack of safe housing, a critical issue, underscores the colonial legacy and the interconnectedness of systems of oppression. As such, it reflects vulnerability across multiple domains ^36^ ^,^ ^37^ ^,^ ^38^ ^,^ ^39^ ^,^ ^40^. Therefore, addressing historical and current structural barriers is critical to ensuring safe, stable, long-term housing conditions. This issue also reinforces discussions about physical access to food, raising concerns about the hygienic and sanitary conditions of places of consumption, as well as the presence of the necessary equipment, as highlighted by the interlocutors of the studies analyzed.

Food insecurity impacted family dynamics among TGNC individuals in multiple ways. Family conflicts intensified, but the common lack of food brought many interlocutors closer to their families ^14^ ^,^ ^41^ ^,^ ^45^. However, stigma and discrimination based on gender and sexual orientation also disrupted family relationships and led to the formation of the “chosen family” - a support network made up of friends and some family members. This network can provide emotional support and physical, food, and financial resources, as well as assistance in obtaining government benefits ^4^ ^,^ ^41^ ^,^ ^45^.

Exploring the “*travesti technologies of (re)existence on the street corner, the lane, and the asphalt*” ^7^ (p. 5; our translation), a study conducted in Brazil demonstrates the production of care among trans people in their search for shelter and support to learn and empower themselves with survival tactics. This is consistent with the mechanisms identified in other studies for overcoming or minimizing hunger. For example, trans people have demonstrated various coping strategies, including personal resilience, forming support networks, and using community restaurants, food banks, and social assistance benefits ^14^ ^,^ ^41^ ^,^ ^45^ ^,^ ^46^ ^,^ ^47^.

Recognizing and valorizing these forms of collective articulation represents a critical intervention in safeguarding the epistemic rights of marginalized communities. Grounded in the premise that “*epistemology is more an ecology of knowledge than an engineering process*” ^62^ (p. 115), we argue that these everyday strategies, with their inherent contradictions and tensions, reaffirm both individual and communal agency as coproducers of knowledge, architects of social transformation, and authors of their own existential narratives. This framework compels a radical reexamination of traditional knowledge-production sites, demonstrating how subaltern epistemologies emerging from marginal lived experiences simultaneously destabilize and enrich dominant epistemological paradigms.

Specifically, the use of food banks and university/community restaurants was identified as an ambiguous strategy. On the one hand, these resources serve as a mechanism to alleviate hunger; on the other hand, they are spaces that can also be permeated by violence, whether symbolic, verbal, or physical. However, these places, designed to accommodate vulnerable populations, can play an important emotional and physical role in alleviating hunger and functioning as a care network ^15^ ^,^ ^41^ ^,^ ^45^ ^,^ ^46^ ^,^ ^47^. This scenario reveals the complexity of the experiences lived by those who depend on these resources to meet their basic needs and underscores the need for sensitive approaches to creating more inclusive and safe environments.

### Limitations

We highlight that all of the studies included in the review were conducted in countries in the Global North, which limits the understanding of trans and nonbinary people’s experiences and perceptions of hunger in different social and historical contexts. This reality exposes enduring structural asymmetries within the global scientific ecosystem, effectively constituting an epistemicide that systematically marginalizes knowledge production from the Global South. The intersection of material barriers (e.g., prohibitive publishing fees); symbolic violence (particularly the hegemony of English as the dominant academic lingua franca); and institutional constraints (chronic underfunding of research infrastructure) not only sustains but actively reinforces neo-colonial academic hierarchies. This self-perpetuating cycle results in the continued epistemological erasure of peripheral scientific knowledge.

We observed that the analyzed studies included distinct populations, making it difficult to analyze the experiences of transgender individuals regarding hunger, indicating that this topic is still under-investigated in qualitative studies.

We also underscore the lack of discussion of race/skin color and ethnicity, which was addressed in depth in only two articles, potentially resulting in an incomplete analysis from an intersectional perspective. In addition, an epistemological superficiality was noted due to the lack of a theoretical foundation provided by the authors, which hinders the robustness of the discussions presented in the study. However, to reduce the possibility of bias in this review, we adopted consistent methodological procedures performed by independent reviewers and assessed the studies that met the eligibility criteria. We also used two tools to assess the quality and methodological rigor of the studies, as well as a tool to determine the level of confidence in the results.

The limitations highlight the need for future research that incorporates a variety of geographical contexts, especially in the Global South, considers intersections of identities, and provides a strong theoretical foundation for a more comprehensive and contextualized understanding of the experiences of food insecurity among TGNC people in order to sensitize public managers to this issue. In addition, this work is unprecedented in that it synthesizes qualitative studies on the relationship between trans people and hunger. By elucidating the connections between food insecurity and gender dissidence, our findings contribute novel insights to the literature and underscore the urgent need for targeted social interventions that advance equity and justice for marginalized gender communities.

## Conclusions

Our findings revealed that TGNC people face high levels of stigma, discrimination, and victimization in multiple settings, including in the family, workplace, and community. In addition, structural factors such as the lack of access to nutritious food, unstable employment, and scarcity of financial resources exacerbate their food insecurity. The analysis shows that food insecurity is linked to issues of race/ethnicity and gender, as well as to experiences of gender-based violence and a lack of safe housing. In particular, forced prostitution is a reality for many trans people due to the social barriers that limit their options, as noted above. The role of support networks, such as the “chosen family”, in mitigating the effects of stigma and food insecurity is also highlighted.

Therefore, by theorizing the nexus between food insecurity and gender dissidence, our analysis makes three critical contributions: it advances intersectional understandings of nutritional inequities; demonstrates how cisnormative food systems produce differential vulnerabilities; and provides evidence-based support for policy interventions that recognize gender self-determination as a food justice imperative. These findings particularly illuminate the embodied consequences of structural violence for gender-marginalized populations, demanding transformative approaches that center queer and trans epistemologies in public health frameworks.

Qualitative research on the relationship between food insecurity and transgender identity is still in its infancy, limiting a more comprehensive analysis. It is therefore recommended that new studies be conducted to explore this association, particularly in light of trans experiences in different regions of the world.

## Data Availability

The sources of information used in the study are indicated in the body of the article.
