# Supplementary material for: Transgender and gender nonconforming people’s vulnerability to food insecurity: systematic review and meta-synthesis
Source: Cad Saude Publica. 2026 Jan 9;41(12):e00058825. doi: 10.1590/0102-311XEN058825 (PMC12799137; doi:10.1590/0102-311XEN058825)
Supplement: Supplementary Material [file 1678-4464-csp-41-12-EN058825-s.pdf]

SUPPLEMENTARY MATERIAL

Table S1 Search strategies.

DATABASE	SEARCH STRATEGIES	REFERENCES
**MEDLINE**	#1 "Transgender Persons"[Mesh] OR (Person, Transgender) OR (Person*, Transgendered) OR (Person, Transsexual) OR (Person, Two-Spirit) OR Transexual* OR Transgender* OR (Transgender Person) OR (Transgendered Person*) OR (Transsexual Person*) OR (Two Spirit Persons) OR (Two-Spirit Person*)<br><br>#2 "Gender Identity"[Mesh] OR (Gender) OR (Gender Identities) OR (Identity, Gender) OR Sexual and Gender Minorities [Mesh] OR (Gender Minorities) OR (Gender Minority) OR (GLBT Person*) OR (lgbtq Person*) OR (LGBT Person*) OR (LGBTQ Person*) OR (Minorities, Gender) OR (Minority, Gender) OR (Person, GLBT) OR (Person, lgbtq) OR (Person, LGBT) OR (Person, LGBTQ) OR travesti*<br><br>#3 "Food Security"[Mesh] OR (Security, Food)<br><br>#4 "Food Insecurity"[Mesh] OR (Food Insecurities) OR (Food Rationing) OR (Insecurities, Food) OR (Insecurity, Food) OR (Rationing, Food)<br><br>#5 "Food Supply"[Mesh] OR (Supply*, Food) OR (Food Supply Chain*) OR (Chain, Food Supply) OR (Supply Chain*, Food)<br><br>#6 "Hunger"[Mesh]<br><br>#7 (((“semi-structured”[TIAB] OR semistructured[TIAB] OR unstructured[TIAB] OR informal[TIAB] OR “in-depth”[TIAB] OR indepth[TIAB] OR “face-to-face”[TIAB] OR structured[TIAB] OR guide[TIAB] OR guides[TIAB]) AND (interview*[TIAB] OR discussion*[TIAB] OR questionnaire*[TIAB])) OR (“focus group”[TIAB] OR “focus groups”[TIAB] OR qualitative[TIAB] OR ethnograph*[TIAB] OR fieldwork[TIAB] OR “field work”[TIAB] OR “key informant”[TIAB])) OR “interviews as topic”[Mesh] OR “focus groups”[Mesh] OR narration[Mesh] OR qualitative research[Mesh] OR "personal narratives as topic"[Mesh]<br><br>#8 #1 OR #2 AND #3 OR #4 OR #5 OR #6 AND #7	369 (#8)
**Web of Science**	#1 (Transgender Persons) OR transgender OR (female to male transgender) OR (gender identity) OR (LGBTQIA+ people)<br><br>#2 (Food Security) OR (Security, Food) OR (Food Insecurity) OR (Food Insecurities) OR (Food Rationing) OR (Insecurities, Food) OR (Insecurity, Food) OR (Food Supply) OR (Rationing, Food) OR (Hunger)<br><br>#3 ALL=((“qualitative research” OR “qualitative method*” OR interview* OR "focus group*" OR ethnographic OR phenomenolog* OR "action research"))<br><br>#4 #1 AND #2 AND #3	60(#34)
**EMBASE**	#1 'female to male transgender'/exp OR 'female to male transgenders' OR 'female to male transsexual' OR 'female to male transsexuals' OR 'FTM transgender' OR 'FTM transsexual' OR 'FTM transsexuals' OR 'trans AFAB' OR 'trans male assigned female at birth' OR 'trans man' OR 'transgender assigned female at birth' OR 'transman' OR 'transmen' OR 'female to male transgender'<br><br>#2 'transgender'/exp OR 'trans people' OR 'trans person' OR 'transgender person' OR 'transgender persons' OR 'transgendered people' OR 'transgendered person' OR 'transgendered persons' OR 'transgenders' OR 'transpeople' OR 'transperson' OR 'transsexual' OR 'transsexuals' OR 'transgender'<br><br>#3 'gender identity'/exp OR 'gender self-identification' OR 'identity, sexual' OR 'sex identification' OR 'sexual identification' OR 'sexual identity' OR 'sexual self-identification' OR 'gender identity'<br><br>#4 'LGBTQIA+ people'/exp OR 'bisexual, gay, lesbian, pansexual, queer, questioning' OR 'gay, bisexual, lesbian, queer, pansexual, asexual' OR 'gay, lesbian, bisexual, pansexual, queer, asexual' OR 'gay, lesbian, bisexual, transgender and intersex' OR 'gay, lesbian, bisexual, transgender and queer/questioning' OR 'gay, lesbian, bisexual, transgender or queer' OR 'gay, lesbian, bisexual, transgender or questioning' OR 'gay, lesbian, bisexual, transgendered and questioning' OR 'gay, lesbian, bisexual, transgendered, queer and questioning' OR 'GLBTI individual' OR 'GLBTI+' OR 'GLBTQ individual' OR 'GLBTQ+' OR 'GLBTQQ' OR 'lesbian, bisexual, queer or pansexual' OR 'lesbian, gay, bisexual or queer questioning people' OR 'lesbian, gay, bisexual, pansexual or queer' OR 'lesbian, gay, bisexual, pansexual, queer and transgender' OR 'lesbian, gay, bisexual, pansexual, queer and/or transgender' OR 'lesbian, gay, bisexual, pansexual, transgender, genderqueer' OR 'lesbian, gay, bisexual, queer and pansexual' OR 'lesbian, gay, bisexual, trans and intersex' OR 'lesbian, gay, bisexual, trans, queer or asexual' OR 'lesbian, gay, bisexual, transgender and intersex' OR 'lesbian, gay, bisexual, transgender and queer' OR 'lesbian, gay, bisexual, transgender and queer/questioning' OR 'lesbian, gay, bisexual, transgender, intersex and queer' OR 'lesbian, gay, bisexual, transgender, intersex or queer' OR 'lesbian, gay, bisexual, transgender, intersex, queer and questioning' OR 'lesbian, gay, bisexual, transgender, intersex, queer/questioning and allied' OR 'lesbian, gay, bisexual, transgender, queer and 2 spirit' OR 'lesbian, gay, bisexual, transgender, queer, 2-spirit, intersex or asexual' OR 'lesbian, gay, bisexual, transgender, queer, intersex and asexual' OR 'lesbian, gay, bisexual, transgender, queer, intersex, asexual' OR 'lesbian, gay, bisexual, transgender, queer/questioning, intersex and allies' OR 'lesbian, gay, bisexual, transgender, queer/questioning, intersex and asexual' OR 'lesbian, gay, bisexual, transgender, transsexual and queer questioning' OR 'lesbian, gay, bisexual, transgender, transsexual, queer, questioning, intersex, asexual, ally, pansexual' OR 'lesbian, gay, bisexual, transgender, two-spirit, and queer' OR 'lesbian, gay, bisexual, transgender/gender diverse, questioning/queer, intersex and asexual' OR 'lesbian, gay, bisexual, transgender/transsexual, intersex and queer/questioning' OR 'lesbian, gay, bisexual, transgendered, queer, questioning, intersex, asexual or two-spirited' OR 'lesbian, gay, bisexual, transsexual, transgender and queer' OR 'lesbian, queer, bisexual, pansexual and asexual' OR 'lesbian/gay/bisexual/transgender/transsexual/queer' OR 'lesbians, gays, bisexuals, transgender, questioning and asexuals' OR 'LGBTI+' OR 'LGBTIAQ' OR 'LGBTIQ+' OR 'LGBTIQA+' OR 'LGBTIQQ' OR 'LGBTQ people' OR 'LGBTQ+' OR 'LGBTQ2' OR 'LGBTQ2S' OR 'LGBTQ2SIA+' OR 'LGBTQA' OR 'LGBTQAI' OR 'LGBTQIA' OR 'LGBTQIA+' OR 'LGBTQIA2S+' OR 'LGBTQPIA+' OR 'LGBTQQ' OR 'LGBTQQIA' OR 'LGBTTQ+' OR 'LGBTTQQIAAP' OR 'LGTBQA' OR 'pansexual, lesbian, queer, bisexual, gay, questioning' OR 'LGBTQIA+ people'<br><br>#5 'gender transition'/exp OR 'gender transitioning' OR 'gender transition'	27 (#11)

	#6 'transsexualism'/exp OR 'female to male transsexualism' OR 'male to female transsexualism' OR 'transsexualism'<br><br>#7 'food security'/exp<br><br>#8 ‘food insecurity'/exp OR 'food insecurity'<br><br>#9 'hunger'/exp OR 'famine' OR 'hungry feeling'<br><br>#10 'semi-structured' OR semistructured OR unstructured OR informal OR 'in-depth' OR indepth OR 'face-to-face' OR structured OR guide OR interview* OR discussion* OR questionnaire* OR (focus AND group*) OR qualitative OR ethnograph* OR fieldwork OR 'field work' OR 'key informant'<br><br>#11 #1 OR #2 OR #3 OR #4 OR #5 OR #6 AND #7 OR #8 OR #9 AND #10 AND [embase]/lim NOT ([embase]/lim AND [medline]/lim)	
**LILACS**	MH:"Pessoas Transgênero" OR (Transgender Persons) OR (Personas Transgênero) OR (Homem Transexual) OR (Homens Trans) OR (Mulher Transexual) OR (Mulher$ Transgênero) OR (Mulher$ não Genética$) OR (Pessoas Trans) OR (Pessoas Transexuais) OR (Pessoas de Duplo Espírito) OR (Terceiro Gênero) OR (Terceiro Sexo) OR Transexuado OR (Transexuais Operados) OR (Transexuais Pré-Operados) OR (Transexuais Pós-Operados) OR (Transexuais não Operados) OR Transexual$ OR (Transexual Feminino) OR (Transexual Pré-Op) OR (Transexual Pós-Op) OR Transgênero$ OR Transvestite OR Tri-Gênero OR Trigênero OR MH:"Minorias Sexuais e de Gênero" OR “Sexual and Gender Minorities” OR “Minorías Sexuales y de Género” OR (Lésbicas, Gays, Bissexuais, Transexuais, Queer, Intersexuais, Assexuais e Outras Identidades) OR (Minorias de Gênero) OR (Pessoas GLB) OR (Pessoas GLBTQ) OR (Pessoas LBG) OR (Pessoas LGB) OR (Pessoas LGBT) OR (Pessoas LGBTQ) OR (Pessoas LGBTQIA+) OR (Queer$)<br><br>MH:"Segurança Alimentar" OR “Food Security” OR “Seguridad Alimentaria” OR (Direito à Alimentação) OR (Direito à uma Alimentação Adequada) OR (Direitos Nutricionais) OR (Garantia de Alimentos) OR (Segurança Alimentar e Nutricional) OR (Segurança Nutricional)<br><br>MH:"Insegurança Alimentar" OR “Food Insecurity” OR “Inseguridad Alimentaria” OR (Racionamento de Alimentos) OR (Racionamento de Comida)<br><br>#4 1 AND #2 AND type_of_study:("qualitative_research"))	12 (#4)
**APA PsycInfo**	#1 {Gender Affirming Care} OR {Gender Expression} OR {Gender Reassignment} OR {Gender Transition} OR {Sexual Minority Groups} OR {Transgender (Attitudes Toward)} OR {Transsexualism} OR {Transgender} OR {LGBTQ} OR {Intersex} OR {Homosexuality} OR {Sexual Orientation} OR {Gender Identity} OR {Pansexuality} OR {Sexual Identity} OR {Transvestism} OR {Two-Spirit} OR {Gender Nonconforming} OR {Gender Nonbinary} OR {Transgender (Attitudes Toward)}<br><br>#2 {Food Deprivation} OR {Hunger} OR {Food Insecurity}<br><br>#3 ((Any Field: (experiences)) OR (Any Field: (interview)) OR (Any Field: (qualitative)))<br><br>#4 1 AND #2 AND #3	2 (#4)
**CINAHL via EBSCO**	#1 (MM "Trans Women") OR (MM "Trans Men") OR (MM "Transgender Persons+") OR "'female to male transgender'" OR (MM "Cross-dressers") OR (MM "LGBTQ+ Persons") OR (MM "Transgender Persons") OR "transgender"<br><br>#2 (MM "Food Security+") OR (MM "Hunger") OR "Food Insecurity"<br><br>#3 ((TI interview OR AB interview) OR (MH “audiorecording” NOT MM “audiorecording”) OR (TI qualitative stud* OR AB qualitative stud*))<br><br>#4 1 AND #2 AND #3	27 (#4)

Table S2 General characteristics of included studies.

REFERENCES	AIM	METHODOLOGY	RESULT
Kirby & Linde, 2020	To explore the knowledge gap regarding nutrition-related health disparities and barriers to adequate nutrition and health maintenance experienced by transgender/GNC college students at a large public university in the southwestern United States.	Participants were college students who identified as transgender or non-binary (NB), aged 18 and over, and who were proficient in English. Recruitment was conducted through community and university organizations using flyers, email announcements, and leader communications. Two study options were offered: a single, in-person interview with the researcher or an anonymous online survey with identical questions. Interviews were conducted privately on campus, audio-recorded for accurate transcription, and lasted one hour or less. Surveys, including the College Student Health Survey (CSS) and the Health Initiative's Voices of Health survey, collected socio-demographic and health habit information. Of the 28 students recruited, data from 26 were analyzed. Qualitative analysis employed systematic thematic analysis of transcripts to identify similarities and differences, with major themes defined by breadth, specificity, content relevance, and overall key findings, while minor themes elaborated on these main findings.	It showed significantly higher rates of food insecurity among transgender/NB students compared to LGBTQ respondents across the Midwest region. Additionally, transgender/NB students in this study reported significantly higher rates of food insecurity compared to the general student population at the same university, including food shortages and lack of money to buy more food. Furthermore, transgender/NB students in this study had nearly double the rates of binge eating compared to the general student population, although these results were not statistically significant. Transgender/NB students in this study also had significantly higher rates of induced vomiting compared to the general student population at the same university.
Russomanno, Patterson & Jabson, 2019	Understanding food insecurity experienced by trans and NB individuals in the southeastern United States.	This study was informed by the constructivist paradigm. Interview questions were intentionally crafted in a semi-structured, conversational tone to allow participants to guide the interview based on their own experiences of food insecurity. An instrumental case study approach was used to focus more on the phenomena being investigated than on individual cases. Intentional sampling with specific criteria was used to recruit members of this hard-to-reach population: English-speaking individuals over 18 years old who identified as TGNC, reported food insecurity issues within the past 12 months, completed the USDA-approved 6-item Food Security Module, and resided in one of the 12 southeastern states of the United States. Informed consent was obtained from all participants before participation in a pre-screening questionnaire assessing food security levels as defined by the USDA Food Security Module. Participants were recruited online through LGBT-focused Facebook groups and advertisements. Each ad included a brief introduction to the study and PI contact information. Twenty participants were interviewed, and two were lost in follow-up between eligibility screening and the scheduled interview. Participants completed a single semi-structured interview either in person or over the phone. Interviews were conducted between April and June 2017, lasted between 30 and 90 minutes, and were audio-recorded. Data collection concluded when saturation was reached. After data collection, qualitative transcripts were entered into NVivo 11 for data management and analysis. Data analysis followed a hybrid method that included an inductive, data-driven approach and a deductive, model-based approach using thematically grounded a priori codes. Four deductive codes (food security, food quality, physical health outcomes, and mental health outcomes) were developed a priori based on known consequences of food insecurity in the general population. Using conventional content analysis, preliminary data analysis began with repeated readings of all data for immersion. Data were then analyzed deductively using a model-based approach according to Crabtree and Miller. Researchers coded all transcripts both inductively and deductively, meeting weekly to discuss coding and resolve any coding discrepancies. Each research team member held various positions, allowing for a nuanced approach to data analysis. The final codebook described four deductive and four inductive codes, including a definition for each code and example quotes from the data. Two overarching themes, "Experiences with Food Security" and "Health Outcomes," emerged from the coded data. A third researcher reviewed the data to ensure that the selected text (data) adequately represented the codes and that the codes were appropriately assigned to the themes.	Participants in our study who identified as TGNC repeatedly described experiencing gender stigma that impacted their financial stability and severely limited their ability to afford adequate food. It is well-documented that TGNC individuals face multi-level discrimination due to their gender identity. Challenges reported by TGNC participants included difficulties finding stable employment with decent pay due to transphobia and gender bias, leading to interpersonal and institutional stressors such as job interview rejections, denied promotion opportunities, and job loss upon coming out as transgender in the workplace. To cope with loss of income and food insecurity, TGNC participants turned to "underground" sources of income, including sex work, which is a documented theme among vulnerable populations experiencing food insecurity. The reliance on sex work to enhance food security is particularly concerning due to the illegality and stigma associated with it, further marginalizing individuals with food insecurity and TGNC identities and increasing the risk of harm. Additionally, TGNC participants accessing food pantries described "stealth" and dressing incongruously with their gender identity to avoid transphobia, resulting in physical discomfort and psychological distress despite facilitating access to local food sources. Moreover, food assistance from the nuclear family was not always a viable option, leading participants to rely on "chosen family" or close friends for food assistance. In the context of a cissexist and binary system of gender identity, where "normal" is defined as "male" or "female," TGNC individuals are viewed as "abnormal" or "less than" due to their transgender or gender non-conforming identity. This gender-based stigma may exacerbate or create feelings of "unworthiness" among TGNC individuals who are also poor and food insecure, potentially explaining why participants in this study felt unworthy of food assistance. The psychosocial consequences of food insecurity are not unique to TGNC individuals; however, for TGNC individuals, each stressor intersected with unique minority stressors stemming from cissexism and transphobia. Multi-level gender-based discrimination and victimization by family members, in the workplace, and within the local food assistance community were layered onto the food insecurity experience of TGNC individuals. Despite facing minority stress and extreme food insecurity, TGNC participants exhibited extraordinary resilience, with resilience being the common thread throughout their experiences, albeit with varying coping strategies.
Sernick et al., 2022	To examine the lived experiences of women living with HIV and food insecurity.	This study is based on qualitative interviews with 64 participants from the SHAWNA (Sexual Health and HIV/AIDS: Longitudinal Assessment of Women's Needs) project. Participants were recruited through peer outreach and the SHAWNA cohort study, using a stratified purposive sampling strategy to ensure representation of WLWH (women living with HIV) from diverse backgrounds, including Indigenous and Black/African WLWH, and those living in both urban and suburban areas of Metro Vancouver. All participants	The participants included 64 WLWH (women living with HIV). Fifty-four were cisgender women, while 10 were assigned male at birth. Of these 10, 3 identified as transgender, 3 as two-spirit, 2 as women, 1 as a heterosexual woman, and 1 as gender queer. Participants' ages ranged from 24 to 68 years, with an average age of 46 years. Thirty-one (48%) participants were identified as Indigenous, 19 (14%) participants were White, 9 (14%) participants were Black/African, and the remaining 5 (8%) participants were of mixed ethnicity or other visible

		were accessing HIV care or living with HIV in the Vancouver metropolitan region and were at least 14 years old. Three experienced interviewers, including two Indigenous WLWH, conducted qualitative interviews from August 2015 to 2017. Audio recordings were transcribed verbatim and checked for accuracy. Data collection and iterative analysis occurred simultaneously, with narratives reported using pseudonyms to ensure confidentiality. Thematic analysis was conducted using ATLAS.ti 7, involving repeated readings of interview transcripts, initial coding based on a priori and emerging themes, and refinement using deductive and inductive methods informed by a modified socio-ecological framework and insights from the literature.	minorities. Our analysis highlighted that the vast majority of participants identified food and nutrition as critical factors in their well-being, health, and self-esteem. Participants were knowledgeable about good nutrition and spoke about the foods they desired, making strong connections between consuming healthy foods and their overall health. However, most faced substantial structural and sociocultural barriers that hindered their food security. Drawing on a socio-ecological framework to center this analysis, two central themes emerged: (a) structural factors hindering food security, including the scarcity of nutritious foods despite the abundance of food services, and (b) sociocultural barriers, such as barriers to accessing culturally specific foods and concerns regarding gender safety in accessing food.
Henry et al., 2022	To explore the meaning and experiences of food insecurity among LGBTQIA+ college students to understand how identity might play a role in those experiences.	The lead researcher, with over seven years of experience in studying college food insecurity, employed an applied ethnographic and qualitative approach, involving LGBTQIA+ UNT students self-identifying as food insecure. Through purposive sampling, participants were recruited via campus-wide emails and faculty announcements, ensuring criteria alignment. Data collection comprised 22 semi-structured Zoom interviews, covering various facets of food insecurity, academic impacts, coping mechanisms, and demographic details. Transcriptions were analyzed using MAXDQA software, with themes deduced from research questions and emergent subthemes identified. Demographic data were quantified for descriptive statistical analysis, offering insights into participants' experiences and perspectives on food insecurity within the college context.	Participants described food insecurity as extending beyond mere access to food, encompassing feelings of anxiety and uncertainty due to diminished family support, financial constraints, and barriers to food access. Coping mechanisms included seeking free food from various sources and managing expenses with affordable fast food, amidst palpable stress over daily sustenance. Adverse effects on mental and physical health were reported, including heightened anxiety and stress, compounded by struggles with pre-existing mental health conditions and physical symptoms such as low energy and impaired concentration. Academic success suffered due to inadequate nutrition and increased work hours, hindering energy levels and concentration. LGBTQIA+ students faced additional hurdles related to familial support, social stigma, and discrimination, often seeking emotional support from peers while professional assistance was less sought. While campus food pantry usage was generally satisfactory, barriers such as mental health issues and lack of transportation hindered access for some. Suggestions for enhancing access to resources included measures to ensure privacy for LGBTQIA+ students and increasing awareness of available support services. External resources encountered stigma and discrimination, prompting LGBTQIA+ students to seek LGBTQIA+-friendly alternatives, influenced by concerns regarding privacy and discrimination. In essence, the study highlights the complex interplay between food insecurity, mental health, social support, and LGBTQIA+ identity among college students, underscoring the need for tailored support services and heightened awareness to effectively address these multifaceted challenges.
Lumens et al., 2024	To examine food insecurity at the intersection of the LGBTQIA2S+ community and the university and college student population, as told by the community itself.	They employed a qualitative, community-based approach involving self-identified LGBTQIA2S+ students at UNCG, utilizing self-selection sampling to ensure diverse experiences. The research comprised two phases: Phase 1 involved photovoice methods to explore environmental factors affecting food access, while Phase 2 included semi-structured interviews to discuss photovoice materials. Due to COVID-19, interviews were conducted via Zoom, lasting 15 to 75 minutes. All data underwent thematic decomposition analysis to identify social themes. Approval was obtained from the University of Alberta's Research Ethics Office.	The first major theme centers on participants' experiences with food insecurity as it relates to their LGBTQIA2S+ identities. While some food access limitations faced by this community are shared with other populations, this section focuses on unique challenges identified by participants. These challenges result from a combination of environmental factors and systemic marginalization, such as passing as cisgender and/or heterosexual, discrimination and microaggressions on campus, support systems, and employment. In addition to this, sub-themes such as spatial opportunities and barriers to food access on and off campus were identified, including welcoming attitudes off campus in downtown Greensboro, lack of full-selection grocery stores on and near campus, negative attitudes off campus, and religious presence off campus. Intersectional factors affecting food security, stigma associated with needing food assistance, questions about mental and physical health, finances, time constraints, and transportation were also identified.

Table S3 Quality assessment of included studies based on CASP score.

Authorship	Year	Country	Interlocutors	Epistemology	Analysis	Methods	CASP score
Kirby & Linde	2020	USA	26 students who identified as TGNC	NA	Systematic thematic analysis	Interviews and surveys	9-A
Russomanno et al.	2019	USA	20 TGNC people	Minority stress theory	Paradigmatic framework of constructivism	Semi-structured telephone interviews	10-A
Ariel Sernick, et al.	2022	Canada	64 WLWH - 7 transgender people	Socio-ecological framework	Iterative thematic analysis	Semi-structured interviews	10-A
Henry et al.	2023	USA	22 LGBTQIA+ - students 12 transgender people	NA	Ethnographic approach	Semi-structured interviews	8-A
Lumens	2022	USA	8 self-identified LGBTQIA2S+ university students: 3 transgender people	Intersectionality and queer theories	Stenner's thematic decomposition analysis	Photovoice and semi-structured interviews	8,5-A
